# Cross-platform comparison for the detection of RAS mutations in cfDNA (ddPCR Biorad detection assay, BEAMing assay, and NGS strategy)

**DOI:** 10.18632/oncotarget.24950

**Published:** 2018-04-20

**Authors:** Jessica Garcia, Julien Forestier, Eric Dusserre, Anne-Sophie Wozny, Florence Geiguer, Patrick Merle, Claire Tissot, Carole Ferraro-Peyret, Frederick S. Jones, Daniel L. Edelstein, Valérie Cheynet, Claire Bardel, Gaelle Vilchez, Zhenyu Xu, Pierre Paul Bringuier, Marc Barritault, Karen Brengle-Pesce, Marielle Guillet, Marion Chauvenet, Brigitte Manship, Marie Brevet, Claire Rodriguez-Lafrasse, Valérie Hervieu, Sébastien Couraud, Thomas Walter, Léa Payen

**Affiliations:** ^1^ Laboratoire de Biochimie et Biologie Moléculaire, Groupe Hospitalier Sud, Hospices Civils de Lyon, Lyon, France; ^2^ University of Lyon, Claude Bernard University, Cancer Research Center of Lyon, INSERM U1052, CNRS UMR5286, Lyon, France; ^3^ Hospices Civils de Lyon Cancer institute, CIRculating CANcer (CIRCAN) program, Pierre Bénite, France; ^4^ Laboratoire Commun de Recherche Hospices Civils de Lyon – BioMérieux, Centre Hospitalier Lyon Sud, Hospices Civils de Lyon, Pierre Bénite Cedex, France; ^5^ Service d’oncologie médicale, Hôpital E. Herriot, Hospices Civils de Lyon, Lyon, France; ^6^ Service de Pneumologie et oncologie thoracique, CHU G Montpied, Clermont-Ferrand, France; ^7^ Université d’Auvergne, UMR INSERM 1240, Clermont-Ferrand, France; ^8^ Service de Pneumologie et cancérologie thoracique, CHU de Saint Etienne, Saint Etienne, France; ^9^ Service d’Anatomopathologie, Groupement Hospitalier Est, Hospices Civils de Lyon, Lyon, France; ^10^ Medical Scientific Affairs, Sysmex Inostics, Inc., Mundelein, IL, USA; ^11^ BioMérieux SA medical diagnostic Department, Centre Hospitalier Lyon Sud, Hospices Civils de Lyon, Lyon, France; ^12^ Univ Lyon, Université Lyon 1, CNRS, Laboratoire de Biométrie et Biologie Evolutive UMR5558, Villeurbanne, France; ^13^ SOPHiA GENETICS SA, Headquarters, CH-1025 Saint Sulpice, Switzerland; ^14^ Service de gastro-entérologie, Hôpital de la Croix-Rousse, Hospices Civils de Lyon, Lyon; ^15^ Hépatogastroentérologie et oncologie digestive, Groupement hospitalier Sud, Hospices civils de Lyon, Pierre Bénite, France; ^16^ EMR 3738 Ciblage Thérapeutique en Oncologie, Faculté de médecine Lyon Sud, Université Lyon 1, Université de Lyon, Oullins; ^17^ Service de Pneumologie aigue spécialisée et cancérologie thoracique, Groupement hospitalier sud, Institut de Cancérologie des Hospices Civils de Lyon, Pierre Bénite, France; ^18^ Service de Biostatistique–bioinformatique et plateforme NGS-CHU Lyon, Hospices Civils de Lyon, Lyon, France

**Keywords:** circulating-free DNA, digital PCR, NGS, liquid biopsy, colon and lung cancer

## Abstract

CfDNA samples from colon (mCRC) and non-small cell lung cancers (NSCLC) (CIRCAN cohort) were compared using three platforms: droplet digital PCR (ddPCR, Biorad); BEAMing/OncoBEAM™-RAS-CRC (Sysmex Inostics); next-generation sequencing (NGS, Illumina), utilizing the 56G oncology panel (Swift Biosciences). Tissue biopsy and time matched cfDNA samples were collected at diagnosis in the mCRC cohort and during 1st progression in the NSCLC cohort. Excellent matches between cfDNA/FFPE mutation profiles were observed. Detection thresholds were between 0.5–1% for cfDNA samples examined using ddPCR and NGS, and 0.03% with BEAMing. This high level of sensitivity enabled the detection of *KRAS* mutations in 5/19 CRC patients with negative FFPE profiles. In the mCRC cohort, comparison of mutation results obtained by testing FFPE to those obtained by testing cfDNA by ddPCR resulted in 47% sensitivity, 77% specificity, 70% positive predictive value (PPV) and 55% negative predictive value (NPV). For BEAMing, we observed 93% sensitivity, 69% specificity, 78% PPV and 90% NPV. Finally, sensitivity of NGS was 73%, specificity was 77%, PPV 79% and NPV 71%.

Our study highlights the complementarity of different diagnostic approaches and variability of results between OncoBEAM™-RAS-CRC and NGS assays. While the NGS assay provided a larger breadth of coverage of the major targetable alterations of 56 genes in one run, its performance for specific alterations was frequently confirmed by ddPCR results.

## INTRODUCTION

*RAS* proto-oncogenes (*HRAS, KRAS* and *NRAS*) encode a family of GDP/GTP-regulated proteins critical for signal transduction mediating cell growth and survival. The four enzymes encoded by the three *RAS* genes are highly homologous, sharing a high degree of identity over the first 90% of proteins [1]. The carboxyl-terminus of the proteins contains a hyper-variable region, which diverges radically in primary sequence from the remainder of these genes. This region of the gene product is susceptible to many post-translational modifications which confer major differences in trafficking and intracellular localization of the mature protein. *RAS* family members are frequently found in their mutated, oncogenic forms in human tumors. Mutant *RAS* proteins are constitutively active, owing to reduced intrinsic GTPase activity and insensitivity to GTPase-activating proteins (GAPs). Overall, activating mutations in *RAS* occur in approximately 20% of all human cancers [2]. Mutations in *KRAS,* mainly in exons 2, 3 and 4 (codons 12, 13, 59, 61, 117, 146), account for nearly 85% of all *RAS* mutations found in human tumors, whereas *NRAS* contributes to ~15%, and *HRAS* to less than 1% [3]. *RAS* somatic mutations are early drivers of tumorigenesis exclusively associated with cancer and therefore provide exquisite specificity for disease. For example, mutations in RAS are prominent drivers of colon, pancreatic and lung cancers.

Epidermal growth factor receptor (EGFR) - targeted monoclonal antibodies (MAb), such as cetuximab and panitumumab, have been used in the treatment of metastatic colorectal cancer (mCRC) since 2004. Numerous studies have demonstrated that RAS mutations are associated with resistance to anti-EGFR antibodies and that approximately 45–50% of CRCs harbor a *RAS* mutation [4, 5]. Based on these findings, anti-EGFR MAbs have been recommended for use in first-line therapy of metastatic CRC (mCRC) for those patients whose tumors are wild-type (WT) for RAS. The determination of RAS mutation status represents the current standard of care for determining mCRC patient eligibility for treatment with anti-EGFR therapy. *KRAS* is also a key biomarker in lung cancer as it is the most common alteration in NSCLC with mutations in *KRAS* occurring in approximately 30% of patients [6, 7]. In NSCLC, 97% of *KRAS* mutations occur in codons 12 and 13 [7]. Mutations in *NRAS* are uncommon in NSCLC (1.1%) [8] while HRAS mutations are extremely rare. *KRAS* is currently not targetable by any approved agents and its occurrence is largely prognostic as it is associated with poor patient outcome [6]. While remaining an elusive therapeutic target, the detection of *KRAS* mutations has diagnostic value as somatic alterations in NSCLC are generally considered mutually exclusive with other driver mutations such as EGFR [9, 10]. Therefore, detecting a *RAS* alteration may aid the clinician since it drastically decreases the chances of detecting another alteration. Testing *RAS* is thus of high clinical importance for mCRC and NSCLC patients, especially in mCRC in which treatment is prescribed according to *RAS* mutation status.

Tumor circulating-free DNA (cfDNA) are small fragments of DNA released into the bloodstream from tumor cells [11]. Mutations in cfDNA may be detected in blood using several techniques, such as: real-time PCR-based assays (Cobas^®^ and Biocartis^®^ assays); digital PCR (dPCR) assays such as droplet-digital PCR (ddPCR) and BEAMing, or next-generation sequencing (NGS) [12, 13]. Detecting mutations in cfDNA offers several distinct advantages when compared to traditional tissue based mutation testing: (i) the sample source for ctDNA testing is blood, which is obtained via a minimally invasive technique and is thus an easily repeatable clinical procedure; (ii) blood sampling and subsequent ctDNA survey at a specific time represent a holistic mutational assessment of the current status of a patient's systemic disease burden, overcoming sampling bias of tissue biopsies performed at a specific disease location. Thus, cfDNA mutational analysis captures spatial and temporal tumor molecular heterogeneity providing a more complete view of the patient's disease status. Many initiatives have emerged over the last few years to assess mutations in cfDNA across a multitude of tumor types [14–16]. The importance of defined sample collection procedures and pre-analytical conditions has been shown to be instrumental for accurate and reliable cfDNA mutation analysis [17]. Our recent work on ctDNA detection in patient samples determined specific pre-analytical considerations to enable a simplification of sample processing thereby streamlining the clinical workflow and ensuring consistency of ctDNA test results for implementation in routine clinical practice [13].

The primary goal of the present study was to compare the performance of three technologies used to detect *KRAS* and *NRAS* somatic alterations in cfDNA from mCRC and NSCLC patients. We evaluated the comparison of results between the molecular profile of FFPE tissues (considered as reference material) and cfDNA samples, and reported on the sensitivity and specificity of each of the cfDNA technologies, which included ddPCR (Biorad), BEAMing (Sysmex Inostics), and NGS using the targeted SWIFT-56G panel (Swift Biosciences). Overall, we demonstrated that the NGS technology provides broader coverage of expanded gene regions which prove useful for exploratory screening of other resistance mechanisms that may occur in addition to those with demonstrated clinical utility. The use of the OncoBEAM^™^ RAS-CRC assay (a BEAMing panel targeting 34 separate *KRAS* and *NRAS* mutations) provided a highly sensitive and accurate detection of RAS mutations, enabling reliable longitudinal monitoring to track the appearance and disappearance of somatic alterations by sampling only plasma-derived cfDNA.

## RESULTS

### CfDNA input

In the two cohorts (NSCLC and mCRC), we observed a correlation between Qubit quantification of cfDNA quantity and the number of WT haploid GE for *KRAS* among samples obtained from mCRC patients (*R* ^2^ = 0.91) and NSCLC patients (*R* ^2^ = 0.86) (Supplementary Figure 1). In these cohorts, the average haploid GE numbers of cfDNA from patients within the CIRCAN cohort was approximately 1200 WT haploid GE/ddPCR reaction (150 WT GE/μL of cfDNA). The basic requirements to appropriately compare the different detection platforms were as follows: (i) 123 μL of cfDNA for the OncoBEAM-TM-RAS-CRC method; (ii) 8 μL of cfDNA/per reaction for the ddPCR Biorad; (iii) and 10 μL of cfDNA for sequencing library preparation (Swift-56G Biosciences) with a cfDNA input quantity ranging between 1 ng and 25 ng. To compare the three assays with the same cfDNA sample and to avoid bias generated by the extraction step, cfDNA was extracted from 4.5 mL of plasma that was eluted in 210 μL of AVE elution buffer. Under these conditions, the concentrations of cfDNA ranged from 0.1 to 9.1 ng/μL (200 to 16,000 WT haploid GE/8 μL) for colon cancer samples, and from 0.1 to 6.2 ng/μL (500 to 1,1000 WT haploid GE/8 μL) for lung cancer samples. These concentrations were sufficient to enable us to carry out the somatic detections for all patients of the cohort. The specificity and sensitivity evaluations are presented in supplementary data (Supplementary Tables 3 and 4). First, we used commercial gold standard from Horizon Diagnostics. secondly, we used expected *KRAS* wild-type patients samples. Since *EGFR* and *KRAS* mutations are mostly mutually exclusive [9], we selected *EGFR* mutated patients to confirm that the BEAMing and NGS assays did not give rise to false-positives cases. The signals are largely below the threshold of positivity (set up at 50 mutated positive signals/reaction for BEAMing and 0.5% for NGS) (Supplementary Table 1).

### Head-to-head performance comparisons of all cfDNA assays with respect to mutational analysis of FFPE samples

Supplementary Tables 2 (mCRC) and 3 (NSCLC) show *KRAS/NRAS* molecular profiles in FFPE reference biopsies (sampled at diagnosis) and in cfDNA using the three different assays. These tables report the: cfDNA concentration per sample (used to assess impact on assay performance); absolute number of mutated and wild-type *KRAS/NRAS* haploid GE for each sample (positive tests are highlighted in red and additional mutations are presented); corresponding mutated allelic fraction (AF); and the *KRAS* and *NRAS* mutation status. Finally, we calculated the concordance rate between each assay compared to FFPE results. For few patients, we found 2 different *KRAS*/*NRAS* mutations in cfDNA.

CfDNA and FFPE biopsies of mCRC were both carried out at diagnosis, enabling paired mutation profile comparisons. In mCRC patients, we found two, four and three mismatches in the ddPCR, the OncoBEAM-TM-RAS-CRC and the NGS assays respectively, compared with FFPE samples (Supplementary Table 2). CIRCAN-colon samples #3 and #23 displayed very low *KRAS* MT-positive signal (59 and 54 absolute count respectively) with the OncoBEAM-TM-RAS-CRC assay and were not detected using the other two assays or in the FFPE samples. CIRCAN-Colon #16 harbored a p.K117D mutation detected with the NGS assay (low level: 1.1%). This mutation is not assessed in the ddPCR and BEAMing panels. The CIRCAN-colon #15 plasma sample harbored a *KRAS* p.G13D mutation and was detected with both ddPCR (22MT haploid GE) and BEAMing (1113 MT-positive signals). NGS assessment also detected this same mutation but at a low allelic fraction (0.8%). FFPE assessment of this sample resulted in a WT determination. In CIRCAN-Colon #21 FFPE, a *KRAS* p.G12V mutation (above positivity threshold) was detected with all cfDNA assays (Supplementary Table 2), though analysis of FFPE was determined to be WT. Using these results, sensitivity was higher for BEAMing (93% vs. 47% and 67% for ddPCR and NGS, respectively), while specificity was lower (69% vs. 76% in both NGS and ddPCR).

In the NSCLC cohort, FFPE biopsy profiling was performed at diagnosis and the cfDNA sampling was performed during disease progression (several months or years after initial diagnosis in some cases). Thus, not all patients had FFPE blocks available for mutation analysis. Among 6 patients with a known WT FFPE sample, 1 was determined to have a p.G12C *KRAS* mutation with BEAMing and NGS (insufficient quantity of cfDNA was available to perform the ddPCR assay). In FFPE samples with known *KRAS* mutations, 6 carried insufficient quantity to perform ddPCR (Supplementary Table 3, ddPCR green box). Finally, 4 *KRAS* mutations were accurately found by ddPCR while 4 samples remained WT (Supplementary Table 3, ddPCR green box).

Fourteen (14) patients were classified as *KRAS* mutant *via* FFPE. Six (6) of these patients lacked sufficient sample volume to perform ddPCR assessment (Supplementary Table 3, ddPCR green box). Of the 8 patients tested by ddPCR, 4 patients were *KRAS* positive while the other 4 were *KRAS* WT (Supplementary Table 3, ddPCR green box). In a WT FFPE specimen, the OncoBEAM-TM-RAS-CRC assay detected one p.G12C *KRAS* mutation (216 mutated signals) in cfDNA that was confirmed by NGS at a low 0.6% allelic frequency (Supplementary Table 3, BEAMing blue box and NGS orange box). There was insufficient cfDNA for ddPCR analysis in this patient. In *KRAS* MT FFPE samples, OncoBEAM-TM-RAS-CRC assay detected a mutation in 8 samples (Supplementary Table 3, BEAMing blue box); while 3 samples remained negative (mutated signal found but under the threshold); and 3 were not covered by the OncoBEAM-TM-RAS-CRC panel. The NGS assay detected mutations in 9 samples (Supplementary Table 3, BEAMing blue box); while 5 samples did not display any mutations (Supplementary Table 3, NGS orange box).

In the lung cancer cohort, we observed 67%, 57% and 64% sensitivity and 100%, 83% and 83% specificity for the ddPCR, BEAMing and NGS assays, respectively. We carried out both OncoBEAM-TM-RAS-CRC and NGS assays on 14 additional samples with unknown FFPE statuses. We found 3 positive cases with the OncoBEAM-TM-RAS-CRC assay, for which only two were confirmed by NGS assay (Supplementary Table 3).

Additional results assessing internal performance and sensitivity of each assay are provided in Supplementary File 2.

### Exploration of concomitant variants in negative and positive *KRAS*/*NRAS* groups in colon and lung cancer

Since the coverage of mutations by NGS sequencing is larger than that provided by digital PCR assays, we analyzed the concomitant presence of additional alterations in our cohort (Supplementary Table 4). We analyzed the Variant Caller Files generated by bioinformatics analyses and we retained all non-synonymous and splice variants found at 1% with at least 50 mutated reads both for *KRAS/NRAS*-positive (Supplementary Table 4A) and negative (Supplementary Table 4B) colon and lung cancer biopsies. In the first group, in addition to the presence of *KRAS/NRAS* mutations, we detected 9 alterations of TP53, PIK3CA and APC at a frequency exceeding 15%. Other genes, including *HRAS* and *PTEN,* yielded alterations at lower frequencies (Supplementary Table 4A). For the second group, we listed 14 genes with somatic alterations. The three main mutated genes with mutational rates exceeding 10% were *EGFR, PIK3CA* and *TP53*. We also observed mutations in tyrosine-kinases, such as *ERBB2* and *ERRB4*, belonging to the same family as EGFR (Supplementary Table 4A).

## DISCUSSION

Prior investigations have shown that cfDNA is detected in almost all patients with advanced cancer [18]. However the low abundance of this tumor-specific genetic material requires highly sensitive techniques to ensure reliable detection for routine practice. The main benefits of utilizing cfDNA as a source of tumor genetic material are based on the safety and convenience associated with minimally-invasive procedures as well as ease of accessibility at any time point. This approach enables clinicians to monitor tumor dynamics and evolution unaffected by sample selection bias. However, accuracy and concordance with tumor tissue mutation testing techniques have not been fully elucidated in patients followed in clinical practices. In this paper, we evaluated the performance of three independent assays to determine *RAS* status in cfDNA using tissue biopsy as reference standard.

Although somatic mutation testing in cfDNA is an area of intense interest, there are still some concerns regarding which assay delivers optimal results for use in the setting of a routine clinical laboratory. Only a handful of studies have approached this issue by comparing several analytical techniques using matched and identical samples [19]. Recently, Bartels *et al*. compared digital PCR (QuantStudio 3D Digital PCR System,ThermoFisher Scientific), NGS (IonTorent, ThermoFisher) and quantitative PCR in 55 cfDNA samples from lung cancer patients and found a 96% concordance when testing for EGFR T790M. The limit of detection for identification of mutations in that study was set at > 0.1% for ddPCR and > 0.2% for NGS. In comparison, we have previously determined that for the analysis of EGFR T790M in cfDNA by ddPCR, the threshold of 0.1% can only be achieved for samples with at least 5000 haploid GE cfDNA input. Below this level, the achieved threshold is only of 1% [13]. In the present study, we determined that a 0.5% allelic fraction represents an adequate threshold for the NGS assay. A lower threshold of positivity can be determined, when the mutation detection of a sample for a specific alteration is carried out by two independent assays (using replicates). Nevertheless, in routine testing generally relying on a single assay to determine mutational status, the threshold of positivity must be increased to avoid a greater risk of obtaining false positives.

Recently, Beije *et al.* [20] and Iwama *et al*.[21] showed that ddPCR performance was more accurate for the detection of *EGFR* mutations in lung cancer and colon cancer than NGS [21]. Interestingly, Beije *et al*. also found that detection of only certain alterations, namely *KRAS*, *PIK3CA* and *TP53* somatic mutations, were consistently more detectable than others also detected in the larger panel (including also *APC, ATM, CREBBP, FBXW7,* and *KMT2D* genes). The concordance between cfDNA and tissue biopsy was higher in patients presenting liver metastasis (55%) than in patients undergoing cfDNA analysis when only the primary tumor was present (39%). This finding may reflect a more robust performance given a higher level of spatial tumor heterogeneity in metastatic cancer patients and underscores the fact that cfDNA represents a more comprehensive surveying of cancer clones that co-exist within the metastatic patient [20]. By contrast, Goldstein *et al*. showed that cfDNA mutation detection in prostate cancers by NGS in the androgen receptor axis may lead to false positive cases that are not observed with ddPCR assays [22]. This highlighted the importance of setting the proper clinical cut-off values specific for the platform being used to detect mutations in cfDNA with particular attention to establishing margins of security that are assay-specific. To our knowledge, only one study performed a cross-comparison of BEAMing with another cfDNA mutation detection method in this setting. In cfDNA samples from lung cancer, both platforms were used to detect EGFR sensitizing mutations as well as the EGFR T790M resistance mutation. In this study, the detection sensitivity of BEAMing was slightly higher than that of the Cobas^®^ EGFR mutation test, Therascreen^™^ EGFR ARMS-PCR and ddPCR^™^ for all three types of EGFR mutations that are clinically-actionable (deletion 19, L858R and T790M). Conversely, specificity was lower with BEAMing compared to the three other platforms [23]. Thus, the present study is the first to cross-compare three different platforms, in two different cancers, using external controls as well as paired samples from blood and tissue. Our results are also in line with those previously reported, namely that BEAMing demonstrates a higher sensitivity than either ddPCR or NGS. Indeed, without increasing background noise (false positive cases), PCR pre-amplification enabled us to detect very low mutant allelic fractions [23]. The enhanced BEAMing sensitivity was further evidenced by the fact that the OncoBEAM-TM-RAS-CRC assay confirmed 93% of patients with a FFPE RAS+ mutation status (we used tissue biopsy samples as previously reported as reference standards for evaluating cfDNA [23, 19, 20]) and even identified positive cases classified as WT through FFPE testing (i.e. resulted in mismatches). These mismatches or “false-positive” cases are defined as somatic alterations detected in cfDNA that are not detected in FFPE analyses. This is common in the liquid biopsy setting and may be attributed to spatial and temporal tumor heterogeneity. With regards to the spatial heterogeneity, tissue biopsy samples originate from single (and often small) biopsies while a cfDNA sample may capture the patient's mutational status more comprehensively by examining the entire tumor burden [20].

The heterogeneity of CRC has been underscored and demonstrated in numerous studies as researchers have highlighted significant differences in the *KRAS* mutational status between primary *vs* metastatic tumors [24–26]. Intra-tumor heterogeneity may lead to the underestimation of the tumor genomic landscape that is provided by testing of single-site tumor biopsy samples. Because plasma receives cfDNA from the various heterogeneous tumor clones in the body, NGS sequencing of cfDNA allows a readily available and non-invasive method for studying and monitoring tumoral heterogeneity and the total tumor burden in the body [27]. In lung cancer, temporal heterogeneity of the tumor may affect the efficacy of particular cancer treatments. Thus, the molecular pattern of tissues sampled at diagnosis may differ from patterns observed in cfDNA using a highly sensitive technique when the cfDNA sample is evaluated in a patient undergoing disease progression.

In our study, the false-positive rate of cfDNA results *vs.* the RAS status reported for lung cancer patient cohort whose tumors were reported as WT using a tissue method ranged from 0% to 17% (Supplementary Table 3) depending on the assay used. The false-positive rate in the colon cancer cohort was higher and ranged from 8% to 31% (Supplementary Table 2). Overall, we observed a very low real rate of false-positive (high specificity) in all three assays. For all assays, specific thresholds of positivity were defined. For the Biorad ddPCR assay, the threshold of positivity was defined as detection of 5 mutated haploid GE/reaction, which translates to acute-off threshold of 0.08% using commercial control DNA. For the OncoBEAM-TM-RAS-CRC assay, the threshold of positivity was 50 mutated positive signals/reaction (according to the manufacturer's determine cut-off threshold) using Horizon's wild-type standard DNA. Finally, for the NGS assay, the threshold of positivity was set up at 0.5% mutant allelic frequency.

Interestingly, the pre-amplification of starting cfDNA material targeting *KRAS*/*NRAS* exons 2, 3 and 4 by multiplex PCR in the OncoBEAM-TM-RAS-CRC assay did not increase the risk of obtaining a false-positive background. Moreover, this assay was able to expose 4 colon cancer and 1 lung cancer patients as true-positives when their FFPE profile was WT, as evidenced by the consistently lower rates of concordance obtained (Supplementary Tables 2 and 3). Secondly, we observed “false-negative” cases (meaning an alteration found in tissue biopsy but not in plasma). This may originate from the limited sensitivity of conventional methods used. Conventional quantitative PCR has a threshold at 1% allelic frequency. This was demonstrated in the Aura study [23], for the T790M mutation, the sensitivity and specificity were 73% and 67%, respectively, with the cobas^®^ EGFR Mutation Test, and 81% and 58%, respectively, with BEAMing dPCR. The lower specificity of BEAMing assay was likely due to the detection of additional positive cases in WT reference samples.

In the present study, 49% of samples examined had less than 1000 haploid GE cfDNA input (Supplementary Figure 1), explaining the necessity for using a highly sensitive method to compensate for the low cfDNA input and low ctDNA release encountered in certain patients. Notably, in case of low input of cfDNA, BIORAD digital PCR assay trended towards to overestimating the allelic mutated frequencies in some cases (CIRCAN-COLON #12 and #16, for example), while the qualitative aspect of positivity of results were in agreement with the other assays. Allelic factions in ddPCR was moderately correlated to those in NGS or BEAMing (R^2^ coefficient 0.64 and 0.68 respectively), but mutated allelic fractions in BEAMing correlated strongly to mutated allelic fractions in NGS (R^2^ coefficient 0.99). Here, we found that the BEAMing assay was the best to detect somatic mutations and thus limit the rate of true false-negative results. Indeed, another explanation of false-negative are the panels used; i.e. a targeted assay would not detect an alteration not covered by the panel but is considered here as a false-negative if the panel used in tissue was wider. The detection panels between assays are very distinct, since the OncoBEAM-TM-RAS-CRC includes 34 frequent RAS (*KRAS* and *NRAS*) DNA alterations, Biorad's assay is restricted to alterations of *KRAS* on exon 2, and the 56G oncology panel provides the overall molecular profile of the patient in one assay [28, 29].

Another explanation for false-negative cases is the copy-number threshold defined to consider a positive sample. Indeed, using our data, we set this threshold at 0.5% for NGS and > 4 haploid GE and > 0.08% of MAF for ddPCR from Biorad and 50 positive mutation signals for the OncoBEAM-TM-RAS-CRC assay (See OncoBEAM RAS CRC IVD instructions for use) [30]. Most mismatches detected *via* the OncoBEAM-TM-RAS-CRC assay had low numbers of mutated signal, which remained undetected using the other two assays, emphasizing the sensitivity of this ddPCR technology. Overall the best matches were obtained with the paired colon cancer samples (FFPE molecular profile and cfDNA sampling conducted at diagnosis) for all three assays, while lung cancer cfDNA samples were collected during disease progression.

Interestingly, when considering BEAMing as the reference standard, the sensitivity of cfDNA NGS was 71%, specificity 88%, PPV 84% and NPV 76%. At this stage of the discussion, this may explain some of the mismatches observed since the profile of patients may have evolved in the case of *RAS*-negative patients or chemotherapy may decrease the proliferation and survival of the mutated *KRAS/NRAS* subclones in RAS-positive patients. Furthermore, mismatches could also be due to (i) the weak release of mutated cfDNA by the tumor (lowering assay sensitivity), (ii) the presence of low proportion of mutated tumoral cells in the samples, (iii) tumor heterogeneity (the tissues used for FFPE molecular profiling may not be representative of the entire tumor), or (iv) the presence of mutated clones in the brain (the release of mutated cfDNA into the blood being restricted in this localization).

Hence, our findings have several strengths but also show some limitations. The main shortcoming is that we did not assess clinical relevance of our molecular results. Indeed, this study aimed only at cross-comparing three different platforms for RAS mutations. Obviously, the next step will be to assess whether detection of certain mutations at low allelic frequencies are clinically meaningful. Another limitation is that our study only included a relatively small cohort of patients (59 in all). Nevertheless, this relatively small cohort is one of the largest groups of patients to be analyzed for cfDNA RAS mutation in cross-platform comparisons [19]. Another drawback of our study arises from the kinetics of blood sampling in the lung cancer cohort (at diagnosis *vs* during progression). Indeed, this difference in sampling may have generated a risk of bias regarding temporal tumor heterogeneity. However, the strengths of our study reside in the comparison of three platforms for cfDNA analysis based on paired samples. To our knowledge, a head-to-head performance comparison of these three cfDNA mutation detection platforms (BEAMing, ddPCR, and NGS) has never been undertaken. Furthermore, most blood samples were compared to their paired tissue samples.

Costs and turnaround time, in routine practice, of these assays are not similar. Thus, these outcomes may be considered. With respect to the two most complementary assays examined here, the BEAMing assay had the quickest turnaround time (2 days) *versus* the NGS assay (1 week), which required more time-consuming steps from library preparation until the time of bio-informatics analysis of the results. The digital droplet PCR assay, although less informative than the two other assays, had the benefit of the shortest turnaround time (8 hours) to complete mutational analysis of *KRAS* exon 2. Concerning the cost, the BEAMing and NGS assays were comparable, whereas the ddPCR assay required only half the cost to perform the analysis versus that of the other two methods.

In conclusion, our findings argue in favor of the use of the Sysmex Inostics BEAMing technology for the detection of somatic *KRAS* and *NRAS* mutations, as a highly specific and sensitive alternative to conventional ddPCR (Biorad). Though, we also highlighted the complementarity between the OncoBEAM-TM-RAS-CRC and NGS combined with the 56G oncology panel, which often confirmed dPCR results and provided a larger overview of the major targetable alterations of 56 genes in one run at diagnosis with a 0.5% threshold. The low detection threshold of the OncoBEAM-TM-RAS-CRC assay (0.03%) is also notable as it reduces the amount of detectable ctDNA (owing to the pre-amplification step) and thus enables clinicians to work from smaller cfDNA concentrations. This technique would thus be interesting to monitor the kinetics of mutated haploid GE numbers for cancer patients under treatment in a minimally-invasive manner, since longitudinal testing of serial tissue biopsy samples is both practical and feasible.

## MATERIALS AND METHODS

### Patients

Samples were collected within the framework of the CIRCAN (“CIRculating CANcer”) study, which is a prospective program established to comprehensively evaluate tumor biomarkers in cfDNA at the Lyon University Hospital. In the present study, we analyzed cfDNA in plasma samples derived from blood from mCRC patients at diagnosis (*n* = 25), as well as metastatic NSCLC patients during disease progression (*n* = 34). All tumor cases were histologically or cytologically confirmed on FFPE biopsy specimens. RAS mutation testing was performed on FFPE for 45 patients (25 mCRC and 20 NSCLC). However, in 14 NSCLC cases, the quantity and/or quality of the FFPE specimen were insufficient for RAS mutation analysis. Patient outcome and demographic data were collected during the course of the study. The extraction, amplification and mutation analyses of cfDNA were performed by investigators who did not have access to or prior knowledge of clinical data and were also blinded to patient outcome following therapy.

### Sample collection

FFPE tumor samples (*n* = 25 for mCRC and *n* = 20 for NSCLC) were microdissected (microdissector LMD2000, Leica, Germany, EU) and DNA was purified from the areas of the samples with the highest percentage of tumor cells using the QIAamp DNA FFPE Tissue kit (Qiagen, Cat No./ID: 56404, Valencia, CA, USA) as per manufacturer's instructions. These samples were then analyzed using a customized Ampliseq library and next-generation sequencing (NGS) on the Ion PGM (Life Technologies, Carlsbad, CA, USA).

Plasma was prepared from 30 mL of blood collected in K_2_ EDTA tubes (BD, 367525, 18 mg). All blood samples were delivered to the laboratory within 24 hours after collection. Detailed pre-analytical considerations have been previously published [13]. The haploid GE corresponds to the haploid Genome Equivalent (330 GE blood DNA for 1 ng/μL cfDNA).

### Droplet-digital PCR for detection of *KRAS* mutations

**Figure 1:**
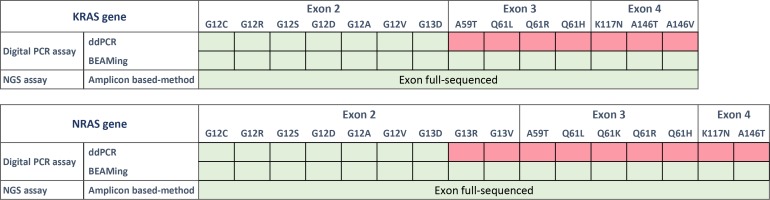
List of the panels provided by Biorad (ddPCR), Sysmex Inostics (OncoBEAM-TM-RAS-CRC) and Swift Biosciences (56G oncology library panel) The “KRAS multiplex kit” screens seven *KRAS* mutations in a single well by ddPCR. NRAS mutations are not included in the panel. The OncoBEAM-TM-RAS-CRC panel enables the detection of 16 *KRAS* mutations and 18 NRAS mutations in exons 2, 3 and 4. Panel of the genes included in the “56G Oncology Panel”. In total, 56 genes (263 amplicons) are amplified and sequenced by NGS using the NextSeq 500 of Illumina. Here, cfDNA samples were analyzed using the three assays while FFPE samples were only tested by NGS.

The sensitive and quantitative QX100 droplet digital PCR system from Biorad (ddPCR, Biorad, Hercules, CA, USA) combines a water-oil emulsion droplet technology with microfluidics (Biorad, 186-3005). All reactions were prepared using the ddPCR Supermix for probes concentrated 2× (Biorad, 186-3024). *KRAS* somatic alterations were detected using a commercial *KRAS Screening Multiplex Kit* provided by Biorad (ref 186-3506). This multiplex ready-to-use detection assay is designed to screen 7 mutations in *KRAS* codons 12 and 13 (Figure 1, labelled in green). Detailed analytical considerations for this assay have been previously published ([13], Supplementary File 1).

### Digital BEAMing for detection of *KRAS* and *NRAS* mutations

OncoBEAM is a highly sensitive and quantitative digital PCR platform utilizing Beads, Emulsion, Amplification and Magnetics (BEAMing). This platform is CE-IVD labelled and produced by Sysmex Inostics (Hamburg, Germany, EU) [31–33]. This assay is based on multiplex PCR targeting somatic alterations which are then followed by a massively parallel second PCR amplification performed on magnetic beads compartmentalized in millions of oil emulsions. Finally, a hybridization step utilizing fluorescent probes specific to wild-type (WT) and mutant (MT) signals is performed with flow cytometry in order to discriminate WT and MT bead populations. Here, we used the OncoBEAM-TM-RAS-CRC^™^ kit (Sysmex Inostics, Hamburg Germany) which enables the screening of 34 somatic genomic alterations in *KRAS* and *NRAS* genes and 30 somatic non-synonymous protein alterations in one run (Figure 1, labelled in green). All experiments were performed according to the supplier's IVD recommendations for clinical application (Instructions for Use, IfU). The pre-specified positivity threshold for each codon was established in a clinical study of 238 patients [30], and on average, the clinical cut-offs provided by the manufacturer were determined to be ~50 mutant beads detected (according to the OncoBEAM RAS CRC kit IfU).

### Targeted next-generation sequencing library preparation

CfDNA libraries were created using the multiple targeted amplicon technology provided by Swift Biosciences according to the manufacturer's instructions (56G Oncology Panel Kit, Swift Biosciences, Ann Arbor, MI, Cat. No AL-56248). Detailed analytical considerations are fully described in the Supplementary File 1. Fastq files, obtained by the demultiplexing of base-call files (BCL), were aligned against the human genome reference Hg19 (GRCh37.p5). Bioinformatics analyses were conducted by Sophia Genetics (Saint-Sulpice). The resulting BAM files were re-aligned for soft-clipping regions to recover potential indels. Variant calls were conducted by comparing the non-reference base against the averaged mean error of the corresponding averaged base quality for a given position. The subsequent Variant Call Files (VCF) were subjected to cross sample background filtering with potential artefacts removed below 3 standard deviations of the mean background noise for each position. Filter criteria for variant calling for absolute mutated allele read counts was set to = > 50 and the depth > 500.

### Ethical considerations

The CIRCAN_ALL study is a prospective observational study conducted at the Lyon University Hospital since December 2015 and intended to comprehensively perform cfDNA mutational analysis in in patients with various types of malignancies. This study was approved by the regional ethics committee Lyon Sud Est IV (CPP L15-188 11/04/2015; amended by L16-160 09/21/2016) and French National committee in Informatics (CNIL 15-131 01.12.2015). Written informed consent for total blood sampling was obtained from all patients included in the study. The study was carried out in accordance with international guidelines and French regulations. All samples and medical data used in this study were anonymized.

### Statistical analysis

Statistics were performed using the latest version of GraphPad InStat software (GraphPad Software, Inc., La Jolla, CA, USA). Normal distribution of continuous variables was assessed with the Shapiro-Wilk normality test. We used the Mann–Whitney test for non-normally distributed variables, and the Student *t*-test for normalized data. A 2-sided *P*-value < 0.05 was considered statistically significant.

## SUPPLEMENTARY MATERIALS FIGURES AND TABLES









## Abbreviations

cfDNA: cell-free DNA
FFPE: Formalin-Fixed Paraffin-Embedded
RT: room temperature
ddPCR: digital droplet polymerase chain reaction
WT: wild-type
EGFR: epidermal growth-factor
NGS: next generation sequencing
