# Supplementary material for: Cross-platform comparison for the detection of RAS mutations in cfDNA (ddPCR Biorad detection assay, BEAMing assay, and NGS strategy)
Source: Oncotarget. 2018 Apr 20;9(30):21122–31. doi: 10.18632/oncotarget.24950 (PMC5940402; doi:10.18632/oncotarget.24950)
Supplement: Supplementary file 1 [file oncotarget-09-21122-s001.pdf]

Cross-platform comparison for the detection of RAS mutations in cfDNA (ddPCR Biorad detection assay, BEAMing assay, and NGS strategy)

SUPPLEMENTARY MATERIALS

Supplementary File 1: Supplementary data for materials and methods

Comparison of ddPCR, BEAMing and NGS panels

Biorad's *KRAS* digital droplet PCR panel is composed of a single PCR reaction that measures the seven most frequently mutated somatic mutations observed in NSCLC and mCRC patients in *KRAS* exon 2. Thus, *NRAS* mutations were not possible to assess with this assay. The OncoBEAM-TM-RAS-CRC™ Panel kit detects 16 *KRAS* mutations and 18 NRAS mutations, enabling the analysis of 34 somatic RAS alterations in a single run. The NGS sequencing panel fully explores exons 2, 3, and 4 of both *KRAS* and *NRAS*, providing the largest breadth of coverage to enable a complete survey of mutations within 263 amplicons of 56 genes, including coverage of 104 exon regions (Figure 1). Similarly, the custom panel employed for the FFPE NGS sequencing fully sequenced exon 2, 3 and 4 of *KRAS* and *NRAS*.

Evaluation of assay sensitivity and specificity

Sensitivity and specificity of the assays were defined as their ability to accurately detect the true-positive and true-negative statuses of *KRAS* and *NRAS* mutations for each sample, compared to results obtained using Horizon reference standards (Horizon Diagnostics, Cambridge, UK) and to paired FFPE samples (used as clinical reference material). Horizon's cfDNA products are all derived from human cell lines, with DNA fragmented to an average size of 160 bp, which mimic the length of DNA fragments observed in plasma cfDNA from patients with cancer.

Sensitivity was also assessed as the ability of each assay to detect a specific fraction of MT/WT signal. For OncoBEAM-TM-RAS-CRC and Biorad's ddPCR method, we used a standard commercial sample of Horizon's Multiplex I cfDNA Reference Standard (Horizon Diagnostics, HD780, Cambridge, UK). This ladder set covers multiple engineered single nucleotide variants/polymorphisms (SNVs/SNPs) with eight mutations at the specific allelic frequencies of 6.3%, 1.3%, and 0.13% (Table S1A). Copy number values measured *via* Biorad's ddPCR on *KRAS* exon 2 are provided with each control. At least three independent assays were performed. Using this reference standard, we were able to: (i) analyze the internal sensitivity and specificity of each assay, (ii) ascertain detection and quantification thresholds, and (iii) routinely assess the performance of each detection assay.

For NGS, the commercial Tru-Q5 DNA reference standard (Horizon Diagnostics, HD732, Cambridge, UK) was used since it is appropriate for all NGS library preparations including targeted amplicon panels. This control covers multiple endogenous SNPs, insertions and deletions and covers multiple engineered mutations at 2.5% (for verified variants). The Tru-Q5 Reference Standard was manufactured using twenty engineered cell lines and pooled to generate a multiple Allelic Frequency multiplex sample (Supplementary Table 1B).

Supplementary File 2: Supplementary data for results

Internal performance and sensitivity of Biorad's ddPCR

To deal with internal specificity, we first assessed the number of MT haploid GE detected in the commercial WT control DNA. The maximum false-positive count was observed with 20,000 WT haploid GE. Here, we found a mean of 10 MT haploid GE (±1.1) associated with a 0.05% allelic frequency false-positive rate inferior to 0.08%. By contrast, when using the 1,000 WT haploid GE control, we found a GE mean of 0.8 (±2.6) corresponding to a 0.08% allelic frequency of false-positive cases (Supplementary Figure 2Ai). In this case the absolute haploid GE count was inferior to the threshold of positivity (≥5 haploid GE), thus classifying it as negative case. Taken together, if any one of the indicators results in a value less than the pre-determined threshold which includes either (i) absolute haploid GE count <5 or (ii) mutated allelic frequency < 0.08%, the sample is deemed negative. We then validated these findings on patient cfDNA samples with known WT *KRAS* in FFPE routine samples (Supplementary Figure 2Aii). Although,

we observed three well-defined mismatches, the maximum false-positive haploid GE determined by ddPCR was 2.8 haploid GE, corresponding to an allelic frequency of 0.3% for the 18 patients of the cohort (including mismatches).

Next, the sensitivity of the assay was investigated using Horizon's commercially available MT controls for a single *KRAS* p.G12D mutation set to 6.3%, 1.3% and 0.1% allelic frequencies. As displayed in Supplementary Figure 2Bi, no meaningful differences were observed between the expected and measured allelic frequencies, although a slight background (around 0.05%) of false-positive cases was obtained with the WT control. This false positive rate amongst WT controls has recently been determined to result from sonication induced oxidative DNA damage resulting in high rates of C > A and G > T transversions when genomic DNA is fragmented with focused ultrasonication (F. Holtrup, Sysmex Inostics R&D). These results were then confirmed in colon/lung cancer patients with a known KRAS mutational status in FFPE samples (Supplementary Figure 2Bii). Among the 23 FFPE biopsy-positive colon and lung cancer samples analyzed, 11 displayed *KRAS* mutations that exceeded the ≥5 MT haploid GE threshold in cfDNA.

Internal performance and sensitivity of BEAMing

The performance of BEAMing was assessed using an identical methodology to that applied to evaluate the performance of ddPCR. Thus, the false-positivity background ranged from 0 to 16 MT haploid GE (0.000-0.028% mutated allelic fraction), much less than the pre-specified 50 haploid GE threshold determined by Sysmex Inostics (Supplementary Figure 3A). We then assessed the BEAMing assay in patients with known WT *KRAS/NRAS* in FFPE samples. Among the 19 specimens that were *KRAS/NRAS* WT in biopsies, 14 cfDNA samples were below the threshold of 50 MT haploid GE and were deemed to have no mutation detected, while a mutation was detected in 5 specimens (+25% additional positive cases) (Supplementary Figure 3B).

Horizon's Multiplex I cfDNA standard set was then used to assess the sensitivity of the OncoBEAM-TM-RAS-CRC assay using two MT allelic frequencies of 1% and 0.1% for one mutation in *KRAS* on codons 12 (p.G12D); and two mutations in *NRAS* in codons 59 (p.A59K) and 61 (p.Q61K) (Figure 1, Supplementary Table 1A). In Horizon's 1% controls, we found allelic frequencies very close to expected values (0.71–0.85%), and similar determinations were made with 0.1% controls (0.08–0.11%) (Supplementary Figure 3A). Furthermore, the absolute number of MT haploid GE detected exceeded the positivity threshold (including for 0.1% controls). Finally, we assessed *KRAS/NRAS* mutation detection with BEAMing in cfDNA samples from patients with *KRAS/NRAS* mutations determined in FFPE. We found 7/29 mismatches (Supplementary Figure 3C), including 4 alterations not covered with the OncoBEAM-TM-RAS-CRC assay.

Internal performance and sensitivity of the NGS assay

We determined the specificity of the NGS assay using a commercial NGS Horizon Tru-Q5 DNA reference standard. We measured the background for three sequenced *KRAS* and *NRAS* exons on wild-type regions of the Horizon control DNA, and obtained a maximum of 0.06% false-positive rate (Supplementary Figure 4Aii). The secured threshold of positivity was defined at 0.5% allelic frequency, avoiding false-positives due to potential sequencing background error rates inherent to NGS technology, even if it is commonly used as a threshold at 2.5% to 5% for FFPE NGS samples. The quality indicator, herein named Q30, was at least superior to 85% of sequenced bases, indicating that the error of sequencing is around 1/1,000 in all of sequencing runs (22). Among the 19 lung and colon cancer patients with negative biopsies, 4 additional *KRAS* mutations were detected (Supplementary Figure 4Aii). Secondly, using the Tru-Q5 DNA gold reference standard, containing *KRAS* and *NRAS* somatic alterations at known allelic fractions, we found very close results between expected and measured values at 2.5% and 25% levels (Supplementary Figure 4Bi).

A

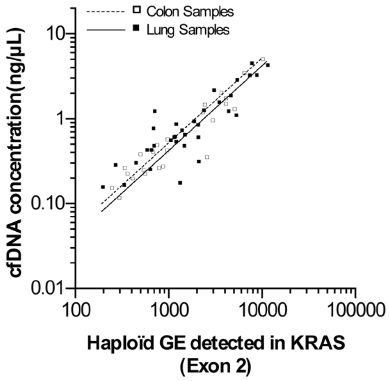

B

Patient	Circulating Free DNA (cfDNA)											
	EGFR			KRAS/NRAS								
	NGS			BEAMing (Sysmex Inostics)					NGS (NextSeq Illumina)			
Patient	Gene	Mutation found	% of mutated cp	Gene	mutated variant	Nb of mutated cp	% of mutated cp	Statut	Gene	Mutation found	% of mutated cp	Statut
CIRCAN-LUNG 07	EGFR	delEX19/T790M	50% / 16%	KRAS/NRAS	WT	11	0.018%	<LD	KRAS/NRAS	WT	0.0%	<LD
CIRCAN-LUNG 13	EGFR	L858R	0.8%	KRAS/NRAS	WT	6	0.011%	<LD	KRAS/NRAS	WT	0.0%	<LD
CIRCAN-LUNG 23	EGFR	L858R	3.5%	KRAS/NRAS	WT	5	0.006%	<LD	KRAS/NRAS	WT	0.0%	<LD
CIRCAN-LUNG 38	EGFR	L858R	3.5%	KRAS/NRAS	WT	9	0.003%	<LD	KRAS/NRAS	WT	0.0%	<LD
CIRCAN-LUNG 41	EGFR	delEX19	2.0%	KRAS/NRAS	WT	1	0.001%	<LD	KRAS/NRAS	WT	0.0%	<LD
CIRCAN-LUNG 58	EGFR	L858R	50%	KRAS/NRAS	WT	16	0.005%	<LD	KRAS/NRAS	WT	0.1%	<LD

Ci

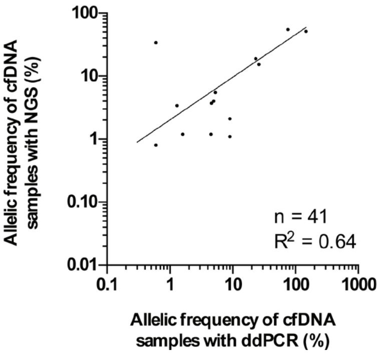

Cii

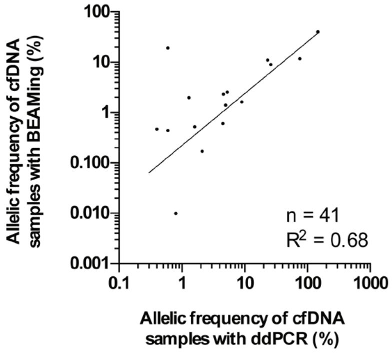

Ciii

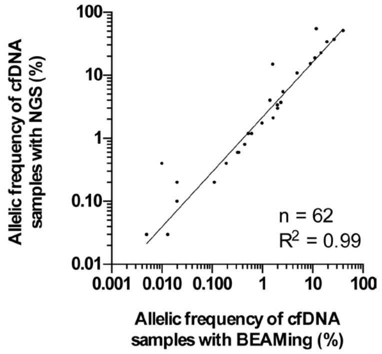

Supplementary Figure 1: (**A**) Correlation between the number of WT haploid GE detected with ddPCR (Biorad) and cfDNA concentration (ng/µl) measured by Qubit method among patients with lung cancer (square full) and colorectal cancer (square empty). (**B**) Analysis of cfDNA carrying EGFR mutations by BEAMing and NGS for the research of KRAS and NRAS mutations. (**C**) Correlation of allelic frequencies found for KRAS and NRAS mutations between ddPCR and NGS (Ci), ddPCR and BEAMing (Cii) and BEAMing and NGS (Ciii). The Rsquared is precised for all comparisons.

Ai

Commercial wild-type DNA				
Expected GE	WT GE	MT GE	%FP	*n*
**20000**	19592.0 (+/- 728)	10.0 (+/- 1.1)	**0.05% (+/- 0.05)**	***5***
**8000**	8672.5 (+/- 549)	4.2 (+/- 2.5)	**0.05% (+/- 0.03)**	***4***
**4000**	4408.0 (+/- 216)	2.7 (+/- 1.7)	**0.06% (+/- 0.04)**	***5***
**1000**	998.9 (+/- 115)	0.8 (+/- 2.6)	**0.08% (+/- 0.16)**	***7***
**0**	0 (+/- 1.1)	0.5 (+/- 1.1)	**0.0% (+/- 0.0%)**	***6***

Bi

Reference standard DNA - Horizon				
	WT GE	MT GE	%	n
**5%MT**	4065.0 (+/- 726)	202.5 (+/- 102)	**5.0% (+/- 1.7)**	**4**
**1% MT**	4920.0 (+/- 1987)	49.0 (+/- 27.9)	**1.0% (+/- 0.3)**	**4**
**0,1% MT**	5440.0 (+/- 2669)	10.1 (+/- 8.5)	**0.2% (+/- 0.05)**	**3**
**WT**	4520.0 (+/- 819)	2.15 (+/- 1.7)	**0.05% (+/- 0.04)**	**4**

Aii

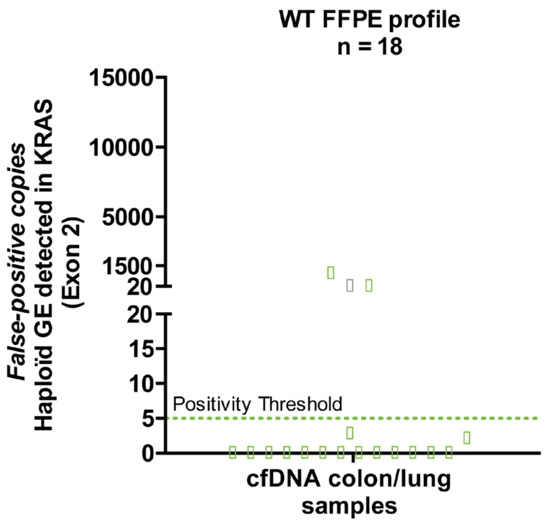

Bii

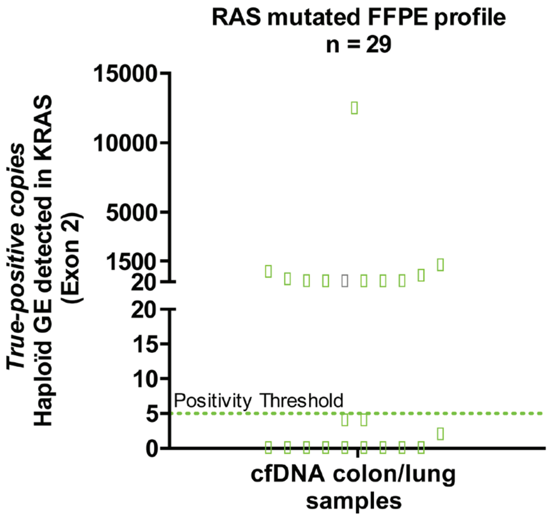

Supplementary Figure 2: Overview of the specificity (**Ai** and **Aii**) and the sensitivity (**Bi** and **Bii**) of the Digital Droplet PCR (ddPCR, Biorad)**.** (A) Determination of false-positive cases (FP) among a range of wild-type DNA haploid GE (Ai) and among cfDNA of colon cancer (CRC) and lung cancer (NSCLC) patients with a negative biopsy status (Aii). (B) Determination of mutated rates of cfDNA in Horizon reference cfDNA standard (Bi) and among cfDNA of patients with positive CRC or NSCLC biopsies. The empty rounds (○) represent true-negative cases, whereas full rounds (●) are true-positive cases. The full rounds (●) in grey are positive cases but the mutation is different comparing of the mutation found in biopsy. All experiments (Ai and Bi) were performed at least 3 times using the "Multiplex Screening KRAS kit" provided by Biorad. The dotted line (Aii and Bii) represents the positivity threshold; n: number of independent experiments; GE: Genomic Equivalent; MT: mutated; WT wild-type; FP: false-positivity.

A

		KRAS						NRAS					
		KR2 Cdn12	KR2 Cdn13	KR3 Cdn59	KR3 Cdn61	KR4A Cdn117	KR4B Cdn146	NR2 Cdn12	NR2 Cdn13	NR3 Cdn59	NR3 Cdn61	NR4 Cdn117	NR4 Cdn146
Horizon 1%	MT Haploïd GE (+/- SD)	583 (+/- 182)	5	5	4	1	16	5	1	587 (+/- 456)	656 (+/- 475)	0	5
	AF% (+/-SD)	0.743%(+/- 0.115)	0.005%	0.002%	0.003%	0.001%	0.018%	0.005%	0.001%	0.851% (+/- 0.117)	0.714% (+/- 0.156)	0.000%	0.007%
Horizon 0,1%	MT Haploïd GE (+/- SD)	66 (+/- 12)	7	4	2	5	13	10	5	78 (+/- 78)	104 (+/- 47)	3	3
	AF% (+/-SD)	0.080% (+/- 0.004)	0.008%	0.004%	0.002%	0.001%	0.011%	0.012%	0.004%	0.095% (+/- 0.029)	0.106% (+/- 0.012)	0.007%	0.005%
Horizon WT	MT Haploïd GE (+/- SD)	7 (+/-6)	4	5	2	1	7	7	7	6	3	2	4
	AF% (+/-SD)	0.011% (+/- 0.004)	0.005%	0.011%	0.004%	0.002%	0.014%	0.026%	0.028%	0.011%	0.004%	0.005%	0.005%

GE : Genome Equivalent
MT : mutated
SD : Standard Deviation
AF : Allelic Frequency

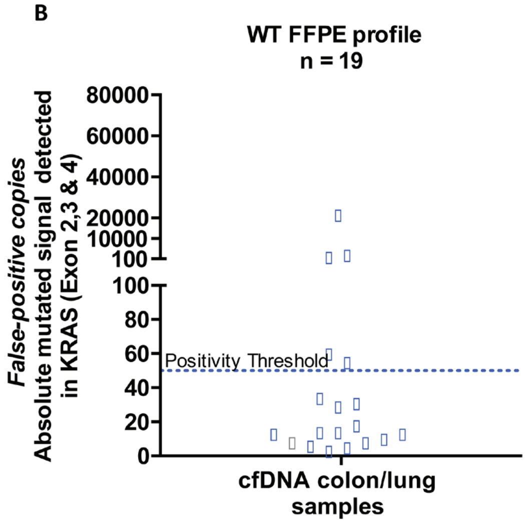

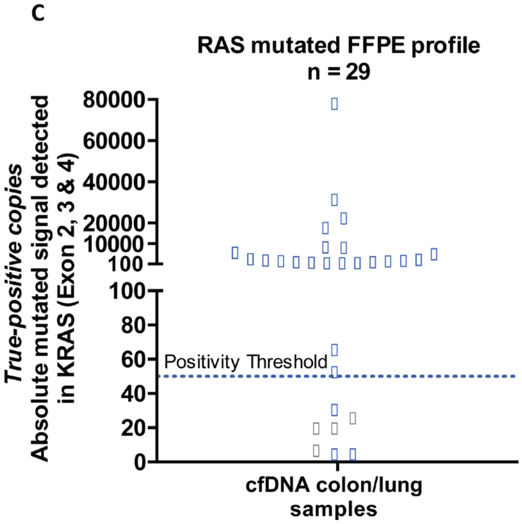

Supplementary Figure 3: Overview of the specificity (**A** and **B**) and the sensitivity (A and **C**) of the Beads, Emulsion, Amplification & Magnetics (BEAMing, Sysmex Inostics) digital PCR. (A) Table representing the absolute number of mutated signals (and corresponding rate of allelic frequency) detected using the Horizon's WT, 0.1%, or 1% reference standard cfDNA. This table shows the specificity (number of MT positive signal detected in reference WT cfDNA) as well as the sensitivity (number of MT positive signal in reference MT cfDNA). (B) Determination of false-positive (FP) cases among cfDNA of lung and colon cancer patients with negative biopsy statuses (assay specificity). (C) Determination of mutated rates among cfDNA of CRC patients with positive biopsy statuses (assay sensitivity). The empty rounds (○) represent true-negative cases, whereas full rounds (●) are true-positive cases. The empty rounds in grey (○) are negative cfDNA cases because the mutation found in biopsy is not explored by the kit. The dotted line (B and C) represents the positivity threshold; n: number of experiments; GE: Genomic Equivalent; MT: mutated; WT wild-type; KR: KRAS mutation; NR: NRAS mutation, Cdn: codon; AF: Allelic Frequency and SD : Standard Deviation.

Ai

Reference Standard DNA Horizon

Gene	Exon	AF% background (+/-SD)
KRAS	2	0.05% (+/- 0.07)
	3	0.04% (+/- 0.07)
	4	0.06% (+/- 0.09)
NRAS	2	0.05 (+/- 0.07)
	3	0.05 (+/- 0.08)
	4	0.06 (+/- 0.08)

AF: Allelic Frequency
SD: Standard Deviation

Bi

Reference Standard DNA Horizon

Gene	Protein	cDNA	AF% Expected	AF% Measured (+/-SD)	n
KRAS	p.G12A	c.35G>C	2.5%	2.27% (+/- 0.48)	28
	p.G12R	c.34G>C	2.5%	2.49% (+/- 0.64)	30
	p.G12V	c.35G>T	2.5%	2.51% (+/- 0.61)	34
	p.G13D	c.38G>A	25.0%	26.96% (+/- 2.42)	19
	p.Q61L	c.182A>T	2.5%	2.28% (+/- 0.72)	19
NRAS	p.Q61K	c.181C>A	2.5%	2.36 (+/- 0.45)	28
	p.Q61L	c.182A>T	2.5%	2.28 (+/- 0.80)	24

AF: Allelic Frequency
SD: Standard Deviation

Aii

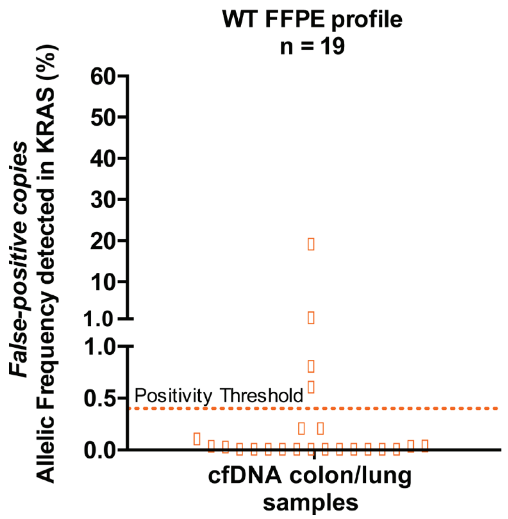

Bii

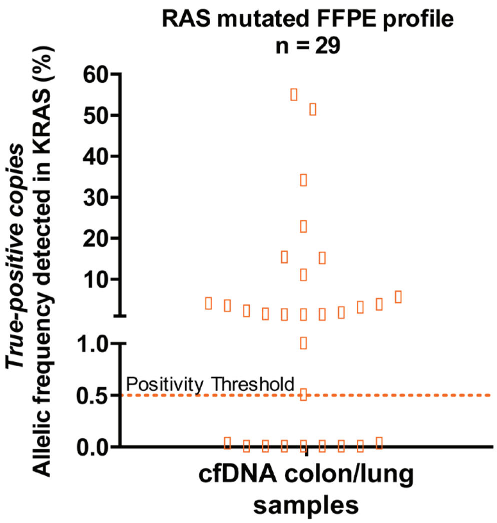

Supplementary Figure 4: Overview of the specificity (**Ai** and **Aii**) and the sensitivity (**Bi** and **Bii**) of the next-generation sequencing (Illumina) and of the 56G oncology panel (Swift Biosciences). (A) Determination of allelic frequency background in the 3 exons of KRAS and NRAS genes among Horizon's reference standard DNA (Ai) and among cfDNA of lung and colon cancer patients with negative biopsy statuses (Aii). (B) Determination of mutated rates of cfDNA in Horizon's reference standard (Bi) and among cfDNA of lung and colon cancer patients with positive biopsy statuses. The empty rounds (○) represent true-negative cases, whereas full rounds (●) are true-positive cases. All experiments (Ai and Bi) were performed at least 9 times. The dotted line (Aii and Bii) represents the positivity threshold; n: number of experiments; AF: Allelic Frequency; SD: Standard Deviation; WT: wild-type.

Supplementary Table 1: (A) Verified mutations of the Horizon's Multiplex I Standard set covers multiple engineered single nucleotide variants with eight mutations at predefined levels of 5%, 1%, and 0.1% allelic frequencies. (B) Verified mutations of the Tru-Q5 Reference standard provided by Horizon Discovery, manufactured using ten engineered cell lines and pooled to generate a 5% allelic frequency multiplex sample

A

Horizon's Multiplex I cfDNA standard set			
**Chromosome**	**Gene**	**Variant**	**Expected allelic frequency, %**
7p12	EGFR	L858R	5.0%–1.0%–0.1%
7p12	EGFR	ΔE746-A750	5.0%–1.0%–0.1%
7p12	EGFR	T790M	5.0%–1.0%–0.1%
7p12	EGFR	V769-D770insASV	5.0%–1.0%–0.1%
12p12.1	KRAS	G12D	6.3%–1.3%–0.13%
1p13.2	NRAS	Q61K	6.3%–1.3%–0.13%
1p13.2	NRAS	A59T	6.3%–1.3%–0.13%
3q26.3	PIK3CA	E545K	6.3%–1.3%–0.13%

B

Horizon Tru-Q5 DNA reference standard			
**Chromosome**	**Gene**	**Variant**	**Expected allelic frequency, %**
2p23	ALK	F1174L	2.5%
7q34	BRAF	V600E	8.0%
7q34	BRAF	V600G	2.0%
7q34	BRAF	V600K	2.0%
7p12	EGFR	G719S	16.7%
7p12	EGFR	L858R	2.1%
7p12	EGFR	T790M	2.1%
10q26	FGFR2	S252W	2.0%
13q12	FLT3	ΔI836	2.5%
9q21	GNAQ	Q209L	2.5%
2q33.3	IDH1	R132C	2.5%
15q26.1	IDH2	R140Q	2.5%
9p24	JAK2	V617F	2.5%
12p12.1	KRAS	G12A	2.5%
12p12.1	KRAS	G12R	2.5%
12p12.1	KRAS	G12V	2.5%
12p12.1	KRAS	G13D	25.0%
12p12.1	KRAS	Q61L	2.5%
15q22.31	MEK1	P124L	2.5%
9q34.3	NOTCH1	L1601P	2.4%
1p13.2	NRAS	Q61K	2.5%
1p13.2	NRAS	Q61L	2.5%
3q26.3	PIK3CA	E545K	2.5%
3q26.3	PIK3CA	H1047R	30.0%

Supplementary Table 2: Matches between colorectal cancer molecular profiles from FFPE biopsies and the cfDNA profiles obtained using all three detection assays, namely Biorad's Digital Droplet PCR (ddPCR), Sysmex Inostics' BEAMing method and Illumina's next-generation sequencing (NGS) combined with Swift Biosciences' 56G oncology panel. See Supplementary_Table_2

Supplementary Table 3: Matches between lung cancer molecular profiles from FFPE biopsies and the cfDNA profiles obtained using all three detection assays, namely Biorad's Digital Droplet PCR (ddPCR), Sysmex Inostics' BEAMing method and Illumina's next-generation sequencing (NGS) combined with Swift Biosciences' 56G oncology panel. See Supplementary_Table_3

Supplementary Table 4: Overview of other mutations found in mCRC and lung cancer patients by Illumina's next-generation sequencing (NGS) using the 56G oncology panel of Swift Biosciences in KRAS/NRAS-positive (A) or -negative (B) cohorts. See Supplementary_Table_4
